# PReS-FINAL-2358: T helper cells in henoch-schönlein purpura/iga vasculitis

**DOI:** 10.1186/1546-0096-11-S2-P348

**Published:** 2013-12-05

**Authors:** B Gülhan, D Orhan, E Karabulut, F Özaltın, R Topaloğlu, A Düzova, S Özen

**Affiliations:** 1 Pediatric Nephrology & Rheumatology, Hacettepe University, Ankara, Turkey; 2 Pediatric Pathology, Hacettepe University, Ankara, Turkey; 3 Biostatistics, Hacettepe University, Ankara, Turkey

## Introduction

Henoch-Schönlein purpura (HSP)/IgA vasculitis is the most common childhood vasculitis in Turkey. During the course of the disease, renal involvement may be observed in 30-60% of patients. In recent years, the role of T helper (Th) cells in pathogenesis of HSP/IgA vasculitis has become a focus for research.

## Objectives

The aim of the study was to investigate the role of Th1, Th2, Th17 and Treg cells in disease patogenesis and their relations with clinical and histopathological parameters.

## Methods

Twenty-two patients diagnosed as HSP/IgA vasculitis with renal biopsy and 6 skin biopsies were included in the study. Non-tumoral renal tissues of nephrectomy materials of 20 Wilms tumor patients and non-pathological skin biopsies of five patients served as the control group. IFN-gamma (Th1), IL-4 (Th2), IL-17 (Th17) and FOXP3 (Treg) were analysed through immunohistochemical staining. Dispersion and intensity scores of immunohistochemistry were evaluated both in the glomerular and tubulointerstitial areas.

## Results

Twenty two Henoch-Schönlein nephritis patients (12 girls, 10 boys) were included in the study. Mean age of the patients was 9,99 ± 3,37 (4,1-15,8) years. At the time of biopsy, 19 patients had microscopic hematuria and proteinuria, two had macroscopic hematuria and one had isolated proteinuria. ISKDC results of the patients revealed: eight patients with grade 2, 12 grade 3a, one grade 3b, and one grade 5.

Renal biopsy specimens of HSP/IgA vasculitis patients had significantly higher IFN-gamma, IL-4, IL-17 glomeruler and tubular scores when compared to the control group. Interferon-gamma staining scores correlated with proteinuria at the time of kidney biopsy. Interleukin-17 glomerular scores correlated negatively with serum albumin levels and positively with proteinuria. Furthermore, IL-17 glomerular scores correlated with the percentage of crescents. HSP/IgA vasculitis group had more FOXP3^+ ^cells on interstitial area when compared to the control group. However, there was no difference in the amount of glomerular and tubular FOXP3^+ ^cells between HSP/IgA vasculitis and the control group. There was no correlation of FOXP3 expression with any clinical parameter.

Skin biopsies of 6 HSP patients and non-pathological skin biopsies of the control group were used for the second part of the study. In the specimens obtained from the area with a lesion, IFN-gamma, IL-4 and IL-17 expressions were statistically higher when compared to normal skin. FOXP3 expressions were not statistically different.

## Conclusion

Our results show the presence of all T helper subtypes in HSP/IgA vasculitis. Relations of Th17 cells with proteinuria and crescent suggests its role in renal prognosis of the patients. Better understanding of the role of these cells and processes may highlight their potentials as therapeutic targets.

## Disclosure of interest

None declared.

